# 472. The impact of sarcopenia on unfavorable outcomes in lung transplant patients with COVID-19

**DOI:** 10.1093/ofid/ofad500.542

**Published:** 2023-11-27

**Authors:** Min Han, Jung Ah Lee, Su Jin Jeong, Jung Ho Kim, Jin Young Ahn, Jun Yong Choi, Nam Su Ku, Joon-sup Yeom

**Affiliations:** Yonsei University School of Medicine, Seoul, Seoul-t'ukpyolsi, Republic of Korea; Yonsei University College of Medicine, Seoul, Seoul-t'ukpyolsi, Republic of Korea; Yonsei University College of Medicine, Seoul, Seoul-t'ukpyolsi, Republic of Korea; Yonsei University College of Medicine, Seoul, Seoul-t'ukpyolsi, Republic of Korea; Yonsei University College of Medicine, Seoul, Seoul-t'ukpyolsi, Republic of Korea; Yonsei University College of Medicine, Seoul, Seoul-t'ukpyolsi, Republic of Korea; Division of Infectious Diseases, Department of Internal Medicine, Yonsei University College of Medicine, Seoul, Seoul-t'ukpyolsi, Republic of Korea; Division of Infectious Diseases, Department of Internal Medicine, Yonsei University College of Medicine, Seoul, Seoul-t'ukpyolsi, Republic of Korea

## Abstract

**Background:**

Coronavirus disease 2019 (COVID-19) in immunocompromised patients has become an important issue owing to the high transmissibility of the Omicron variant of severe acute respiratory syndrome coronavirus 2. Sarcopenia in lung transplant patients is associated with a poor prognosis, and lung transplant patients are more vulnerable to COVID-19. However, the effect of sarcopenia on lung transplant patients with COVID-19 remains unclear. This study aimed to investigate the effect of sarcopenia on lung transplant patients with COVID-19 and the risk factors for sarcopenia following COVID-19 in lung transplant patients.

**Methods:**

We performed a retrospective cohort study of lung transplant patients with COVID-19. Sarcopenia was defined as being within the lower 25% of the thoracic muscle index based on cross-sectional area measurements of the pectoralis, paraspinal, serratus, and latissimus muscles at the fourth vertebral using computed tomography (CT). Multivariable logistic analysis was conducted to identify the factors associated with sarcopenia in lung transplant patients following COVID-19.

**Results:**

Of the 445 patients receiving follow-up care after lung transplantation, 81 were diagnosed with COVID-19. The median age was 59.0 (interquartile range 48.0–64.0) years, and 55 (67.9%) patients were male. Among patients within three years of lung transplantation, the sarcopenic group had a significantly higher rate of hospitalization (100.0% and 46.9%, *P* = 0.006) and oxygen demand (63.6% and 18.8%, *P* = 0.016) than the non-sarcopenic group. Multivariable analysis showed that body mass index before COVID-19 [odds ratio (OR), 0.77; confidence interval (CI), 0.58–0.96; *P* = 0.040] and serum albumin (OR, 0.12; CI, 0.01–0.79; *P* = 0.047) were associated with sarcopenia after COVID-19.

**Table 1.**
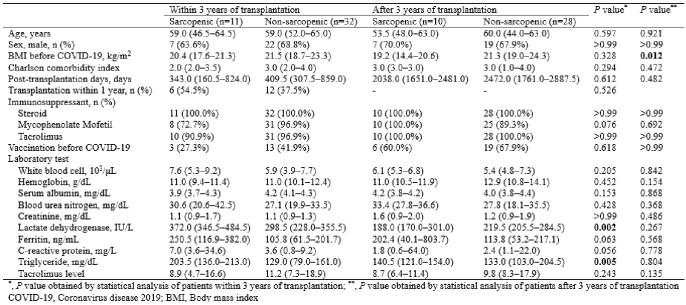
Comparing characteristics of lung transplant patients according to pre-COVID-19 sarcopenia status

**Table 2.**
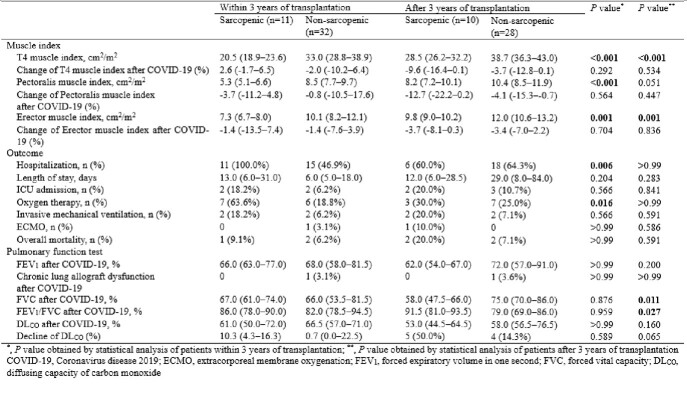
Comparing muscle index and outcome of COVID-19 in lung transplant patients according to pre-COVID-19 sarcopenia status

**Figure:**
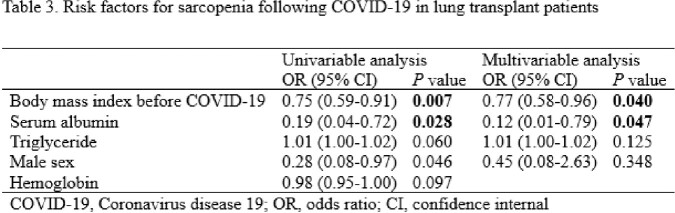

**Conclusion:**

Sarcopenia was associated with poor outcomes of COVID-19 in patients within three years of lung transplantation. Low albumin levels and body mass index were identified as risk factors for post-COVID-19 sarcopenia. Assessments and interventions for sarcopenia are needed to improve the prognosis of lung transplant patients with COVID-19.

**Disclosures:**

**All Authors**: No reported disclosures

